# Defining Optimal Nutrition Behaviors to Determine Benefit–Cost Ratio of Federal Nutrition Education Programs

**DOI:** 10.3390/nu17193076

**Published:** 2025-09-27

**Authors:** Annie J. Roe, Andrea Leschewski, Shelly Johnson, Joey Peutz, Kristin Hansen, Siew Guan Lee, Jocelyn Elvira, Nurgul Fitzgerald

**Affiliations:** 1 Margaret Ritchie School of Family and Consumer Sciences, University of Idaho, Moscow, ID 83844, USA; khansen@uidaho.edu; 2 Ness School of Management and Economics, South Dakota State University, Brookings, SD 57007, USA; andrea.leschewski@sdstate.edu; 3 University of Idaho Extension, Northern District, Coeur d’Alene, ID 83814, USA; sjohnson@uidaho.edu; 4 University of Idaho Extension, Southern District, Caldwell, ID 83605, USA; joeyp@uidaho.edu; 5 University of Idaho Extension, Central District, Twin Falls, ID 83301, USA; siewguanl@uidaho.edu; 6 Project ECHO Idaho, University of Idaho, Boise, ID 83702, USA; 7 Department of Nutritional Sciences, Rutgers, The State University of New Jersey, New Brunswick, NJ 08901, USA; nurgul.fitzgerald@rutgers.edu

**Keywords:** expanded food and nutrition education program, supplemental nutrition assistance program education, cost–benefit, nutrition, chronic disease, adult, optimal nutrition behavior, automated self-assessment 24 h dietary recall, food and physical activity questionnaire

## Abstract

**Background/Objectives**: Historically, federal investment in nutrition education programming in the U.S. has exceeded USD 500 million annually. The purpose of this study was to develop evidence-based Optimal Nutrition Behavior (ONB) criteria related to data collected by federal nutrition education programs and apply these criteria to established cost–benefit analysis methodology to determine the healthcare savings attributable to participation in these programs. **Methods**: A quasi-experimental study was conducted using the Eat Smart Idaho (ESI) program as a model for federal nutrition education programs (*n* = 78) and a matched control group (*n* = 78). Surveys administered at baseline and post-program collected dietary intake and physical activity behaviors. Optimal Nutrition Behaviors were defined as those behaviors that were associated with reduced chronic disease risk as determined by published meta-analyses, systematic reviews, or large cohort studies. Direct and indirect benefits generated by ESI were calculated using established methods. **Results**: The frequency of individuals meeting ONB criteria at post-assessment was significantly greater in the ESI group compared to control for all diseases except for colorectal cancer (*p* ≤ 0.05). ESI’s benefit–cost ratio of $11.62 suggests each dollar spent to administer the program results in USD 11.62 of economic benefits through chronic disease medical costs and lost earnings avoided. **Conclusions**: Federal funds supporting nutrition education programs contribute to reduced medical costs.

## 1. Introduction

United States (U.S.) land-grant universities apply research to develop evidence-based community programming. Through partnerships with federal, state, and local governments, programs are implemented as part of the Cooperative Extension System. Extension has long been interested in identifying the impacts and public value of its programming efforts. University of Idaho Extension’s Eat Smart Idaho (ESI) program is a nutrition education program that helps individuals and families make healthy food choices and lead active lifestyles. The program provides interactive, evidence-based information to assist people in improving their overall well-being. Through classes, presentations, recipe demonstrations, educational displays, and online resources, ESI builds self-efficacy of participants to develop the knowledge and skills needed to create healthier habits. Eat Smart Idaho also includes social marketing campaigns and efforts to improve policy, systems, and environmental changes in communities. Eat Smart Idaho is supported through two United States Department of Agriculture (USDA) grants, the Supplemental Nutrition Assistance Program Education (SNAP-Ed) [1], and the Expanded Food and Nutrition Education Program (EFNEP) [2].

Several studies have been conducted to quantify the public value of similarly funded nutrition education programs. A foundational study nearly 25 years ago by Lambur et al. [3] and Rajgopal et al. [4] provided initial methodology for a cost–benefit analysis for EFNEP at the state level [3,4]. This study measured changes in food-related behavior using 24 h food recall and a food practice checklist with 14 basic food-related behaviors. Utilizing these standard EFNEP evaluation tools, they provided the groundwork for quantifying the relationship between diet and chronic disease risk. In this study, the authors introduced the term Optimal Nutrition Behaviors (ONB) to define the prevention criteria based on the data collected from EFNEP evaluation tools. Between 2002 and 2017 this methodology has been used, with slight variations in data collection approaches, to assess the benefit–cost ratio of several other EFNEP and SNAP-Ed programs [5,6,7,8,9]. Since these studies, new evaluation tools have been adopted by EFNEP.

In 2017, the food intake assessment methods went through a substantial change. The 20-item Food and Physical Activity Questionnaire (FPAQ) was adopted nationally to evaluate EFNEP participant behaviors. EFNEP leaders from 14 states developed and validated FPAQ questions focusing on five domains: diet quality, physical activity, food safety, food security, and food resource management [10,11]. In addition to a new survey to collect behavior changes, a standardized tool for collecting 24 h dietary intake data was also piloted during this same time for use in EFNEP [12]. The Automated Self-Administered 24-h Dietary Assessment tool (ASA24^®^) is a free online tool used to collect and analyze 24 h dietary recall data [13]. Since these extensive changes took place in EFNEP networks nationwide, there has not been a study that applies the Rajgopal et al. [4] methodology to data collected using these new evaluation tools.

The primary purpose of this study was to develop evidence-based Optimal Nutrition Behavior criteria related to data collected by FPAQ and ASA24^®^. The secondary purpose of this study was to apply these criteria to established cost–benefit analysis methodology to determine the healthcare savings and lost earnings avoided attributable to participation in the Eat Smart Idaho program.

## 2. Materials and Methods

### 2.1. Subjects and Study Design

A quasi-experimental study was conducted in the federal fiscal year 2019 (FFY2019; October 2018–September 2019) to evaluate the cost–benefit of the Eat Smart Idaho program (ESI; Idaho SNAP-Ed and EFNEP). Study subjects were adult participants (aged 18 years and above) recruited in Idaho’s four ESI districts in Northern, Southern, Central, and Eastern District Extension. Participants eligible for ESI programming were eligible for the study. The ESI audience is individuals and families with low-income who are eligible for federal food assistance programs, such as the Supplemental Nutrition Assistance Program (SNAP), the Emergency Food Assistance Program (TEFAP), the National School Lunch Program, and the Special Supplemental Nutrition Program for Women, Infants, and Children (WIC). Study subjects were recruited from low-income housing sites, schools with 50% or greater free and reduced lunch participation rates, low-income health clinics, and local food pantries.

Subjects were recruited for intervention or control groups at matched locations. Both groups took pre- and post-surveys 6 weeks apart to collect demographic data, exposure to ESI’s policy, systems, and environmental programs, FPAQ, and ASA24^®^. The intervention group received a six-lesson series from the curriculum *EFNEP’s Families Eating Smart and Moving More* [14] during the 6 weeks between the pre-and post-surveys. The control group received no education.

### 2.2. Data Collection Instruments

#### 2.2.1. Demographic Questionnaire

Subjects’ demographics, including self-reported age, sex, race, ethnicity, highest level of education, residence location, monthly household income, and family composition (number of people living in the household and ages of the children living in the household), were collected using surveys administered upon enrollment (Supplementary Materials).

#### 2.2.2. Food and Physical Activity Questionnaire

Food and nutrition-related behaviors were collected using a validated Food and Physical Activity Questionnaire (FPAQ) [15], taken at entry and exit (Supplementary Materials). The FPAQ consists of 20 questions with scaled responses ranking how frequently a participant performs food and nutrition-related behaviors, which concentrate on five domains (i.e., diet quality, physical activity, food safety, food security, and food resource management). Questions use 6-point to 8-point frequency scales. Participants indicated the frequency of their behavior based on the number of days in a week or on a scale from never performing the behavior, rarely (about 20% of the time), sometimes (40%), often (60%), usually (80%), or always. Data from diet quality, physical activity, and food safety questions were used in this study.

#### 2.2.3. Dietary Intake Assessment

The Automated Self-Administered 24-H (ASA24) dietary assessment tool, version 2018, developed by the National Cancer Institute, Bethesda, MD was used to record foods and beverages consumed [13]. The online ASA24^®^ was administered during the pre- and post-evaluation. The assessment was accessed through iPads and the use of hotspots or the facility’s Wi-Fi to access the tool. Each subject was provided with a unique username and password to complete the assessment confidentially while allowing for pre- and post-data to be matched.

#### 2.2.4. Assessment of Exposure to Policy, Systems, and Environmental Projects

All subjects completed a survey to capture exposure to ESI’s policy, systems, and environmental projects (Supplementary Materials). Subjects were asked if they had accessed any of the food pantries or cared for children at any of the schools where ESI implemented Healthy Pantry or Smarter Lunchrooms Movement (SLM) programming. They were prompted to recall whether they had noticed the food pantry environmental and policy changes (MyPlate signs, food donation lists) as well as the Long Live Idaho (LLI) social marketing campaign posters displayed in their community.

### 2.3. Nutrition Education

*EFNEP’s Families Eating Smart and Moving More* was delivered in a six-lesson series [14]. This evidence-based curriculum is designed for adults with low-income to improve nutrition, physical activity, food safety practices, and food resource management. The intervention group participated in this series taught by an ESI Nutrition Instructor over six weeks. Each one-hour session included a lecture style lesson accompanied by a PowerPoint presentation, hands-on activities, a recipe demonstration, a tasting component, and take-home handouts. The lesson topics included the following:

Lesson 1—Choosing to Move More Throughout the Day
Lesson 2—Choosing More Fruits and Vegetables
Lesson 3—Fix it Safe
Lesson 4—Plan: Know What’s for Dinner
Lesson 5—Shop: Get the Best for Less
Lesson 6—Shop for Value, Check the Facts

### 2.4. Defining Optimal Nutrition Behaviors

#### 2.4.1. Chronic Diseases and Assessment Variables

Previous cost–benefit studies were used to identify diet- and physical activity-related diseases [3,7,8]. Diseases included in the analyses were as follows: colorectal cancer, heart disease, stroke, hypertension, osteoporosis, type 2 diabetes, obesity, and foodborne illness.

Questions from the FPAQ and nutrients from the ASA24 were assigned to each disease. In addition to the evidence from the literature, the collective expertise and experience of the research team was used to select the FPAQ questions that assessed behaviors related to risk of the specific disease. For dairy, whole grains, micronutrients, and Healthy Eating Index, ASA24 data were used. When similar variables appeared in both instruments, we utilized the data from validated FPAQ. Because the FPAQ included assessment of fruit and vegetable intake, these food groups were not included in the ASA24 data utilized in order to prevent double-counting.

#### 2.4.2. Disease Risk Related to Diet or Physical Activity Variables

To determine the specific food intake and physical activity levels in relation to the risk for selected diseases, PUBMED and Google Scholar databases were searched for meta-analyses or systematic reviews which were published from 2014 to 2022, using the following search terms: disease AND risk AND [FPAQ or ASA24 Variable]. If a systematic review or meta-analysis was not available, a search was conducted for primary research articles.

Strength of evidence was rated as strong if a systematic review or meta-analysis provided evidence supporting reduced risk related to the specific FPAQ or ASA24 variable. If there was not a systematic review, but there were primary research articles that included large sample sizes, or national datasets, evidence strength was rated as emerging. If there were no supporting articles, if the primary research was pilot data, if there were small sample sizes, or if they did not have strong findings, the evidence was rated as weak. Items with strong or emerging evidence were included in the Optimal Nutrition Behavior (ONB) criteria. Items with weak evidence were not included.

#### 2.4.3. Setting Criteria for Optimal Nutrition Behavior

The Dietary Guidelines for Americans (DGA) [16] and the Physical Activity Guidelines for Americans (PAGA) [17] were primarily used to determine the cutoff points on the FPAQ questions associated with the ONB. For the ASA24 variables, ONB criteria were set using the DGA when assessing food group items and the Dietary Reference Intakes (DRI) [18,19] when assessing specific nutrients. The ONB criteria for both the FPAQ and ASA24 variables were also compared to the meta-analyses, systematic reviews, and original research articles identified to ensure that there was not a different dose of food group or nutrient that was found to be related to reduced risk of a specific disease. If a specific amount was identified, this was used instead of the general recommendation from the DGA, PAGA, or DRI. 

Individuals meeting the ONB criteria on more than 50% of the disease-specific items were recorded as achieving the ONB for that disease. Individuals meeting the ONB criteria on 50% or fewer of the items assigned to a particular disease were recorded as not achieving the ONB for that disease. Similar mid-point cutoff levels have been used in the other literature when determining the adherence to a dietary pattern score [20,21].

### 2.5. Cost–Benefit and Statistical Analyses

Following the approach outlined in Rajgopal et al. (2002) [4], identified ONB improvement was then used to estimate the direct and indirect benefits generated by ESI through the prevention or delay of the eight identified chronic diseases and conditions. *Direct benefits* represented the chronic disease medical costs avoided due to ONB improvement. Described in detail in Rajgopal et al. (2002) [4], direct benefits per graduate [*E*] for each chronic disease were estimated as $\left[A\right]\times \left[B\right]\times \left[C\right]\times \left[D\right]=\left[E\right]$, where $\left[A\right]$ was the disease incidence rate for low-income females, [*B*] was the share of the disease attributable to diet, [*C*] was the disease ONB improvement attributable to ESI, and [*D*] was the present value of medical costs avoided due to delay or prevention of the disease. Indirect benefits per graduate [*G*] represented the lost earnings foregone through chronic disease prevention or delay due to ONB improvement and were estimated as $\left[A\right]\times \left[B\right]\times \left[C\right]\times \left[F\right]=\left[G\right]$, where [*F*] was the present value of lost earnings foregone, and [*A*]–[*C*] were as previously defined. The estimates of [*A*], [*B*], [*D*], and [*F*] were obtained from the literature [22]. Minimum wage was used to reflect low-income populations and generate a conservative estimate of earnings, which could also approximate conditions in rural settings. Number of days lost to illness was based on general U.S. population data. For each disease, estimates of [*C*] were obtained using probit regression analysis in which the dependent variable was a binary indicator of ONB improvement. If there was not a significant difference between the control and intervention, changes to risk of that condition could not be attributable to Eat Smart Idaho and the condition was not included in the cost benefit calculation. Model covariates included the intervention, ESI, as well as demographic characteristics (age, sex, race, ethnicity, educational attainment, household composition, and rural versus urban location). Predicted probabilities were calculated post probit estimation to isolate the effect of ESI on ONB improvement for each disease. Total benefits per graduate were then calculated by summing direct and indirect benefits per graduate for each chronic disease and condition.

Direct costs of administering ESI during FFY2019 were obtained from program records and included salaries, benefits, recruitment, office space, utilities, equipment, supplies, training, overhead, travel, contracts and agreements, and a 17% marginal excess burden, representing the economic loss to society from taxation. Total benefits per graduate were then compared to direct costs per graduate using a benefit–cost ratio. A benefit–cost ratio greater than one indicates the economic benefits generated by ESI exceed program costs, while a ratio less than one indicates costs exceed benefits. Sensitivity analyses were undertaken to assess robustness of results to discount rate variation.

Differences between the intervention and control groups’ characteristics were assessed using independent *t*-tests for the continuous variables and chi-square tests for the categorical variables. Within-group differences comparing the pre- and post-assessments in median ONB scores were conducted using analysis of variance (ANOVA) (SAS proc GLM) to compare means and McNemar’s test or chi-square to compare frequency counts within and between groups, respectively. These analyses were conducted using SAS software, version 9.4 of the SAS System for Windows. Copyright © 2002–2012 by SAS Institute Inc., Cary, NC, USA.

## 3. Results

### 3.1. Subjects

Through partnerships with 250 community organizations, ESI was implemented in 31 of Idaho’s 44 counties, and 1096 adults participated in the direct education class series. A subset of these individuals participated in the cost–benefit research study. Subject characteristics are summarized in Table 1. The average age of the intervention group was slightly higher than the control group (*p* = 0.03). Although both groups were mostly female, there was a significant difference in sex between the two groups (*p* = 0.03). No other statistically significant differences were identified between groups. Both groups were primarily White and not Hispanic/Latino, mostly with a high school diploma/GED or higher education, and from more populated areas.

### 3.2. Optimal Nutrition Behaviors

The Optimal Nutrition Behavior (ONB) criteria for each chronic disease or condition assessed are outlined in Table 2. Criteria with strong evidence are indicated by green, and emerging evidence is indicated by orange. All conditions included variables from both the FPAQ as well as the ASA24, with the exception of foodborne illness, which only included the four questions from the FPAQ. The number of diet- or physical activity-related items for each disease were 13 for colorectal cancer, 16 for heart disease, 11 for stroke, 9 each for hypertension and osteoporosis, 12 each for type 2 diabetes and obesity, and 4 for foodborne illness.

Table 3 summarizes the distribution of disease-specific ONB criteria met by groups at pre- and post-assessment. Out of a possible maximum total ONB criteria score of 86, median numbers achieved were 22.5 at pre- and 24 at post-assessment for the control group, while the intervention group’s scores increased from 24 at pre- to 34 at the post-assessment.

Within each of the control and intervention groups, subjects who started with a higher score (i.e., in the upper quartile) at the pre-assessment made greater improvements in their scores in comparison to the participants who started with scores in the lowest quartile. For example, the total disease risk score increased by 1 and 5 points in the control group but 8 and 12 points in the intervention group for the 25th and 75th percentiles, respectively.

Analysis of variance (SAS, proc GLM) indicated no statistically significant differences (*p* > 0.05) in average number of ONB criteria met between groups at pre-assessment for all diseases or conditions; at post-assessment, significantly higher ONB criteria were met for all conditions in the intervention group compared to the control group (*p* ≤ 0.05). Further, there was a significant increase in post scores compared to pre scores for all diseases or conditions within the intervention group but not the control group (*p* ≤ 0.05).

The frequency of individuals meeting the criteria for ONB at pre- and post-assessment is summarized in Table 4. Chi-square analyses indicated that there was no statistically significant difference between groups at the pre-assessment for any disease or condition. At the post-assessment, the number of individuals meeting the ONB criteria was significantly higher in the intervention than the control group for all diseases and conditions; the difference was only not statistically significant for colorectal cancer. Although greater numbers of individuals met the ONB criteria at post-assessment than pre-assessment for most of the diseases, these differences reached statistical significance only for colorectal cancer, obesity, and type 2 diabetes in the control group. More individuals met the ONB criteria at post-assessment compared to pre-assessment for all diseases in the intervention group (McNemar’s test; *p* ≤ 0.05).

### 3.3. Policy, Systems, and Environmental Interventions

The exposure to PSE interventions is summarized in Table 5. Chi-square analysis identified a significant difference in exposure to Smarter Lunchrooms Movement (SLM) schools at baseline, with more individuals in the control group being involved with an SLM school than in the intervention (*p* ≤ 0.05). More individuals in both groups reported recalling MyPlate signs and the Long Live Idaho water poster at post-assessment compared to pre-assessment. There was also a significant difference in the control group’s recall of the Long Live Idaho motion poster from pre- to post-assessment. There was a decrease in the intervention groups’ exposure to pantry interventions from pre- to post-assessment.

### 3.4. Benefit–Cost Ratio

Cost–benefit analysis results presented in Table 6 indicated that ESI generated USD 9044.58 in total benefits per graduate through improvements in ONB for chronic disease prevention. Program costs per graduate for FFY2019 totaled USD 778.24. For the intervention group (*n* = 78), this equates to USD 705,477.08 in benefits generated at a cost of USD 60,703.04. Extrapolating to all ESI graduates in FFY2019, program benefits were estimated to be USD 7,832,606.28, with program costs totaling USD 673,959.37. Eat Smart Idaho’s benefit–cost ratio of 11.62 suggests each dollar spent to administer the program results in USD 11.62 of economic benefits through chronic disease medical costs and lost earnings avoided. Sensitivity analysis results indicated that the benefit–cost ratios ranged from 9.93 to 19.82. Further sensitivity analyses were conducted to determine the benefit–cost ratio assuming only 25%, 50%, or 75% of participants retained ONB improvements long-term. The resulting benefit–cost ratios were all greater than one, at 2.91 for 25% retention, 5.81 for 50% retention, and 8.72 for 75% retention. 

## 4. Discussion

This study developed evidence-based Optimal Nutrition Behavior criteria related to self-reported behavior change data collected by FPAQ and ASA24. This set of criteria was applied to established cost–benefit methodology to determine the healthcare savings and lost earnings avoided that are attributable to participation in federal nutrition education programs such as EFNEP and SNAP-Ed. Eat Smart Idaho, funded through EFNEP and SNAP-Ed, was used as a model for this study. Applying Optimal Nutrition Behavior (ONB) within a cost–benefit framework provides a practical tool for evaluating nutrition education programs. By demonstrating measurable behavior change and economic value, this approach can provide research-based evidence to support sustaining and expanding such programs, particularly in the context of limited federal resources and budget constraints.

Results of this study estimated that every dollar spent on administering ESI resulted in USD 11.62 of economic benefits through avoidance of chronic disease medical costs and lost earnings. This benefit–cost ratio of USD 11.62:1.00 is slightly higher than prior estimates in the literature, which range from USD 0.82:1.00 to USD 10.75:1.00 [5,6,8,9,175]. Benefit–cost ratio heterogeneity likely reflects differences in the programs evaluated, as well as methodological differences across the studies. Notably, this study included a control group, estimated the benefits using updated, evidence-based ONB by utilizing more comprehensive FPAQ variables, and excluded benefit estimation for two infant diseases that were considered in prior analyses. Results suggest that prior estimates obtained using fewer ONB criteria and no control group may have underestimated the economic value generated by EFNEP and SNAP-Ed through chronic disease prevention.

For the development of the ONB criteria, this study utilized the evidence from current national guidelines, systematic reviews, meta-analyses, and large cohort studies indicating reduced risk of diseases or conditions in relation to food intake or physical activity behaviors. Similarly to diseases and conditions explored in previous studies [3,4,5,6,7,8,9], the ONB criteria in this study included risk for colorectal cancer, heart disease, stroke, hypertension, osteoporosis, type 2 diabetes, obesity, and foodborne illness. Unlike the previous studies [3,4,5,6,7,8,9], the current study did not include “infant diseases” and low birthweight or assign different criteria from the 24 h dietary recall for pregnant or nursing individuals, as these conditions were not applicable to most of the ESI participant base. Although there is evidence to support that nutrition and physical activity have the potential to reduce risk of dementia [176,177,178], we excluded dementia because the available data on age of onset and age of death were not conducive to conducting the cost–benefit analysis. Perhaps a focus on a specific disease, such as Alzheimer’s or other neurodegenerative diseases with an earlier age of onset, rather than all-encompassing “dementia” would be an option for future cost–benefit analysis studies to explore. In the current study, tooth decay was also excluded because there were only two questions on the FPAQ that assessed relevant behaviors (i.e., sugar sweetened beverage consumption) with evidence to reduce risk [179].

The current study revealed that the numbers of criteria met by those in the upper and lower quartiles, as well as the average number of ONB criteria met (i.e., meeting >50% of the ONB criteria for any one disease) were very low (Table 3). Individuals in the intervention group significantly improved their numbers of ONB criteria met in comparison to the control group. A remarkable finding was that those who started with a higher score improved more even in the intervention group. These results, coupled with the low range of individuals meeting the ONB criteria, overall indicate a potentially high vulnerability to the disease states that were included in this study. These results also point to great potential to improve the disease risk profile for this population with limited resources. The individuals starting at a poorer health status or lower ONB criteria profiles may require additional support or resources to achieve ONB.

Since the inclusion of policy, systems, and environmental approaches in the SNAP-Ed guidance in the FFY2014 [180], these approaches have been key contributors to SNAP-Ed programming across the nation, reaching individuals and communities on multiple levels of the social ecological model. Because the PSE exposure could act as a confounder when looking into potential effects of the ESI lesson series, we examined but did not find meaningful PSE exposure differences to explain the ONB score differences between control and intervention groups. The control group was exposed to a lunchroom PSE approach more than the intervention group, but their ONB scores remained lower than those of the intervention group. Despite these results, our previous ESI surveys and reports from other states suggest that PSE exposure is likely to influence food intake behaviors. For example, our survey in FFY2019 found that 65% (48 out of 73) of food pantry patrons agreed or strongly agreed that signage (as part of PSE interventions) helped them choose foods from all food groups, demonstrating the potential for PSE to influence behavior change (unpublished data). Similarly, a California SNAP-Ed report indicated that PSE reach predicted positive changes in dietary intake of adults living in SNAP-Ed-eligible households [181]. Another study assessing Utah SNAP-Ed programming found that greater exposure to multi-level programming, including direct education, PSE, and social marketing, was reflective of increased positive dietary behavior changes [182]. The differing conclusions between these PSE findings and our current results could stem from the methodological differences such as variations in the survey questions and locations, and dosage of PSE exposure. The impact of PSE interventions on individual behavior change has not been assessed in previous cost–benefit studies. Because of the potential for PSE approaches to impact ONB, PSE should be considered as a covariate when conducting future cost–benefit assessments of federally funded nutrition education programs with larger populations or programs with additional PSE interventions. Additionally, designing PSE interventions that aim to change or influence behaviors identified in the ONB criteria could allow evaluation of their independent contributions to behavior change and cost-effectiveness, complementing the impact of direct education programs.

As noted earlier, some of the strengths of this study involve its inclusion of a control group, and using a strong evidence base from current systematic reviews, meta-analyses, and national guidelines. Furthermore, this study used an expanded set of food intake and physical activity instruments to determine the ONB criteria. Previous studies utilized a food practice checklist rather than the FPAQ [3,4,8,175]. The food practice checklist contained fewer questions, resulting in fewer ONB or risk factor criteria for each disease state. For example, previous studies using the food practice checklist and a 24 h food recall had 5-6 criteria for ONB related to colorectal cancer [8,175], while using the FPAQ and ASA24 data in the current study included 13 criteria for colorectal cancer. Although these differences make it difficult to align the current study with the methods used previously, current methods provide a significantly expanded and updated set of standards for ONB. As the evaluation methods and questionnaires may continue to change in the federally funded nutrition education programs, future studies will need to take these changes into consideration in future cost–benefit analyses.

The demographic characteristics of participants in this study were similar in income and education level to the other EFNEP and SNAP-Ed programs but lacked diversity in gender, race, and ethnicity [183,184]. Additionally, this sample was drawn from a population in a rural western state. Therefore, generalizability of the results to other populations with different background characteristics is limited.

## 5. Conclusions

This study identified food intake or physical activity behaviors associated with reduced risk of chronic disease and developed evidence-based Optimal Nutrition Behavior (ONB) criteria related to variables collected by federally funded nutrition education programs, such as Eat Smart Idaho (ESI). These criteria were applied to established cost–benefit analysis methodology in a quasi-experimental study to determine the economic benefit of participation in these types of programs. Diseases and the number of diet- or physical activity-related variables included in the assessment were colorectal cancer (13), foodborne illness (4), heart disease (16), hypertension (9), obesity (12), osteoporosis (9), stroke (11), and type 2 diabetes (12). Achieving ONB for each disease was defined as adhering to the recommendations for more than 50% of the relevant variables. Participation in ESI was positively associated with meeting the ONB criteria for all disease states except colorectal cancer. Cost–benefit analyses indicated that every dollar spent on administering the ESI resulted in USD 11.62 of economic benefits through avoidance of chronic disease medical costs and lost earnings. Federal funds supporting nutrition education programs (e.g., EFNEP and SNAP-Ed) contribute to reduced medical costs.

## Figures and Tables

**Table 1:** Subject demographics and other characteristics.

		Control		Intervention		
Variable		*n*	Mean ± SD or%	*n*	Mean ± SD or%	*p*-Value
Age (years)		78	43.0 ± 14.6	78	48.2 ± 14.8	0.03
Monthly income (US dollars)		66	1486 ± 1082	63	1685 ± 1518	0.39
Number of children in the household		78	0.9 ± 1.4	78	1.2 ± 1.5	0.32
Number of people in the household		78	2.9 ± 1.7	78	3.2 ± 1.8	0.37
Sex	Female	57	73	68	87	0.03
	Male	21	27	10	13	
Race	White	72	92	72	92	0.34
	American Indian or Alaska Native	5	7	3	4	
	Asian	0	0	1	1	
	Two or more races	1	1	0	0	
	Not provided	0	0	2	3	
Ethnicity	Hispanic/Latino	20	26	16	21	0.47
	Non-Hispanic/Non-Latino	58	74	61	78	
	Not Reported	0	0	1	1	
Highest level of education	Some high school	4	5	5	6	0.83
	High school or GED	29	37	30	38	
	Some college	26	33	24	31	
	Graduated 2-year college	4	5	6	8	
	College graduate	9	12	8	10	
	Postgraduate	1	1	3	4	
	No response	2	3	0	0	
Residence location	Farm	6	8	4	5	0.91
	Town under 10,000/rural non-farm	15	19	15	19	
	Town/city 10,000–50,000	39	50	37	48	
	Suburbs of cities over 50,000	5	6	5	6	
	Central cities over 50,000	13	17	17	22	

**Table 2:** Evidence base for optimal nutrition behavior criteria.

Food and Physical Activity Questionnaire	ONB Criteria	CC	FBI	HD	HTN	OB	OST	STK	T2D
How many times a day do you eat fruit?	≥2 times/day	[23,24,25]		[26,27,28,29,30]	[31,32,33,34]	[35,36,37,38,39]	[40,41,42]	[26,27,30,43,44,45]	[46,47,48,49,50,51,52]
How many times a day do you eat vegetables?	≥3 times/day	[23,25]		[26,27,28,29,30,45]	[31,32,33,34]	[38,39,53,54,55]	[40,41,56]	[26,27,28,30,43]	[46,47,48,49,50,51,52]
Over the last week, how many days did you eat red and orange vegetables?	≥4 days/week	[57]		[26,58]				[30,58,59]	[60]
Over the last week, how many days did you eat dark green vegetables?	≥2 days/week	[61]		[26,29,62,63]		[64]		[30,62]	[65,66]
How often do you drink regular sodas (not diet)?	≤1–3 times/week			[67]		[68]		[27,67]	[30,47,49,50,69,70,71]
How often do you drink fruit punch, fruit drinks, sweet tea, or sports drinks?	≤1–3 times/week	[72]		[27,30,67]		[54,69,71,73]		[27,67]	[30,47,49,50,69,70,71]
In the past week, how many days did you exercise for at least 30 m?	≥4 days/week	[74,75,76,77,78,79,80]		[81,82,83]	[84,85,86,87,88,89,90]	[83,91,92,93,94,95]	[96,97]	[82,98,99]	[46,100,101,102,103,104,105]
In the past week, how many days did you do workouts to build and strengthen your muscles?	≥2 days/week			[106]	[107,108,109,110]	[111,112]	[96,97,113]		[100,102,104,106,114,115]
How often do you make small changes on purpose to be more active?	≥60% of time	[79,80,116,117]		[118,119,120,121,122]	[123,124,125,126]	[54,92,127,128]		[129]	[100,101,119,122]
How often do you wash your hands with soap and running water before preparing food?	≥80% of the time		[130,131,132]						
After cutting raw meat or seafood, how often do you wash all items and surfaces that come in contact with these foods?	Always		[130]						
How often do you thaw frozen food on the counter or in the sink at room temperature?	≤20% of the time		[130]						
How often do you use a meat thermometer to see if meat is cooked to a safe temperature?	≥80% of the time		[130]						
**24-H Dietary Recall**									
Whole grains	3 oz/day	[23,133,134,135,136,137,138]		[27,30]	[32]	[35,38,64,139,140,141]			[30,46,47,49,50,101,142,143]
Dairy	3 cups/day	[23,137,144]		[145,146]	[32,147]	[148,149]	[150,151]	[43,152]	[47,152]
Seafood	≥8 oz/week			[27,30]		[38]		[27,43]	[51]
Fiber	≥25 g/day	[135,144,153,154,155,156,157]		[30]					
Sodium	≤2300 mg/day			[30,158,159]	[160,161,162,163,164]		[165]	[166]	
Potassium	≥4700 mg/day			[30,159]	[167]				
Calcium	≥1200 mg/day	[137]					[168]		
Vitamin D	≥25 mcg/day						[169]		
Folate	≥400 mg/day	[144]							
Vitamin C	≥90 mg/day						[170,171]		
Healthy Eating Index	≥74.1	[172]		[173]		[174]			

ONB = Optimal Nutrition Behavior; CC = colorectal cancer; FBI = foodborne illness; HD = heart disease; HTN = hypertension; OB = obesity; OST = osteoporosis; STK = stroke; T2D = type 2 diabetes; green = strong evidence; orange = emerging evidence.

**Table 3:** Distribution of the optimal nutrition behavior criteria scores and number of criteria met for each disease or condition.

Disease or Condition	#ONB Possible Maximum Score	Control								Intervention							
		Pre				Post				Pre				Post			
		Mean ± SD	Q1	Mdn	Q4	Mean ± SD	Q1	Mdn	Q4	Mean ± SD	Q1	Mdn	Q4	Mean ± SD	Q1	Mdn	Q4
CC	13	3.1 ± 1.9	2	3	4	3.6 ± 2.3	2	3	5	3.4 ± 2.0	2	3	5	4.7 ± 2.4 *^,†^	3	4.5	7
FBI	4	2.2 ± 0.9	2	2	3	2.2 ± 1.02	2	2	3	2.2 ± 1.0	2	2	3	2.8 ± 1.1 *^,†^	2	3	4
HD	16	4.4 ± 2.0	3	4	6	4.9 ± 2.2	3	4	6	4.5 ± 1.9	3	4	6	6.2 ± 2.3 *^,†^	5	6	8
HTN	9	1.9 ± 1.4	1	2	3	2.2 ± 1.5	1	2	3	1.7 ± 1.3	1	2	3	3.0 ± 1.6 *^,†^	2	3	4
Obesity	12	3.6 ± 1.8	2	3	4	4.0 ± 2.0	3	3	5	3.8 ± 1.7	3	4	5	5.2 ± 2.0 *^,†^	4	5	7
OSTEO	9	1.9 ± 1.4	1	2	3	2.2 ± 1.4	1	2	3	1.9 ± 1.4	1	1	3	2.8 ± 1.6 *^,†^	2	3	4
Stroke	11	3.9 ± 1.7	3	3.5	5	4.2 ± 1.7	3	4	5	4.0 ± 1.6	3	4	5	5.2 ± 1.9 *^,†^	4	5	7
T2D	12	3.7 ± 1.9	2	3	5	4.1 ± 2.0	3	4	6	4.0 ± 1.7	3	4	5	5.4 ± 2.1 *^,†^	4	5	7
Overall	86	24.5 ± 11.2	16	22.5	30	27.4 ± 12.3	17	24	35	25.5 ± 10.8	17	24	32	35.3 ± 13.4 *^,†^	25	34	44

ONB = Optimal Nutrition Behavior; SD = standard deviation; Mdn = median; CC = colorectal cancer; FBI = Foodborne Illness; HD = heart disease; HTN = hypertension; OSTEO = osteoporosis; T2D = Type 2 Diabetes; Overall = total ONB for all diseases or conditions combined. * Significant difference between group means for the same assessment period (ANOVA; SAS proc GLM; *p* ≤ 0.05). ^†^ Significant difference in means from pre (within group) (ANOVA; SAS proc GLM; *p* ≤ 0.05).

**Table 4:** Number and percentage of subjects meeting optimal nutrition behaviors.

Disease or Condition	Control, *n* (%)		Intervention, *n* (%)	
	Pre	Post	Pre	Post
Colorectal Cancer	4 (5)	12 (15) ^†^	5 (6)	20 (26) ^†^
Foodborne Illness	25 (32)	27 (35)	32 (41)	50 (64) *^,†^
Heart Disease	3 (4)	5 (6)	2 (3)	17 (22) *^,†^
Hypertension	4 (5)	6 (8)	1 (1)	15 (19) *^,†^
Obesity	5 (6)	9 (12) ^†^	6 (8)	25 (32) *^,†^
Osteoporosis	3 (4)	3 (4)	4 (5)	14 (18) *^,†^
Stroke	15 (19)	19 (24)	14 (18)	34 (44) *^,†^
Type 2 Diabetes	6 (8)	12 (15) ^†^	9 (12)	26 (33) *^,†^

*n* = 78 per group. * Significant difference in frequency counts between groups (chi-square; *p* ≤ 0.05). ^†^ Significant difference in frequency counts from pre (within group) (McNemar’s test; *p* ≤ 0.05).

**Table 5:** Individuals reporting exposure to policy, systems, or environmental (pse) interventions.

PSE Variable	Control, *n* (%)		Intervention, *n* (%)	
	Pre	Post	Pre	Post
Visit Pantry with PSE Interventions	36 (46)	32 (41)	34 (44)	28 (36) ^†^
Viewed MyPlate Signs	16 (21)	23 (29) ^†^	12 (15)	18 (23) ^†^
Viewed Healthy Food Drive Flyer	21 (27)	20 (26)	25 (32)	18 (23)
Involved in Smarter Lunchrooms Movement School	16 (21)	13 (17)	6 (8) *	7 (9)
Recalled Long Live Idaho Water Poster	11 (14)	23 (29) ^†^	16 (21)	25 (32) ^†^
Recalled Long Live Idaho Play Time Poster	11 (14)	13 (17)	18 (23)	19 (24)
Recalled Long Live Idaho Motion Poster	10 (13)	18 (23) ^†^	10 (13)	17 (22)
Recalled Long Live Idaho Rainbow Poster	15 (19)	22 (28)	16 (21)	18 (23)

*n* = 78 per group. * Significant difference in frequency counts between groups (chi-square; *p* ≤ 0.05). ^†^ Significant difference in frequency counts from pre (within group) (McNemar’s test; *p* ≤ 0.05).

**Table 6:** Eat Smart Idaho cost–benefit analysis.

Direct Benefits Per Graduate	USD 8621.12
Colorectal Cancer ^a^	USD 0
Foodborne Illness	USD 139.43
Heart Disease	USD 90.41
Hypertension	USD 548.22
Obesity	USD 1965.89
Osteoporosis	USD 313.77
Stroke	USD 399.12
Type 2 Diabetes	USD 5164.27
Indirect Benefits per Graduate	USD 423.46
Colorectal Cancer ^a^	USD 0
Foodborne Illness	USD 5.07
Heart Disease	USD 19.80
Hypertension	USD 77.68
Obesity	USD 164.38
Osteoporosis	USD 19.43
Stroke ^b^	USD 0.00
Type 2 Diabetes	USD 137.10
Total Benefits per Graduate	USD 9044.58
Costs per Graduate	USD 778.24
Benefit–Cost Ratio	11.62

^a^ Colorectal cancer was excluded from benefit calculation as regression results indicated the intervention did not have a significant effect (*p* < 0.10) on ONB improvement for the disease. ^b^ Indirect benefits for stroke are USD 0.00 as its average age of onset exceeds the average retirement age.

## Data Availability

The raw data supporting the conclusions of this article will be made available by the authors on request.

## Supplementary Materials

The following supporting information can be downloaded at: https://www.mdpi.com/article/10.3390/nu17193076/s1, Form S1: Participant entry and exit forms; Form S2: Food and Physical Activity Questionnaire; Form S3: PSE programs survey.

## Abbreviations

The following abbreviations are used in this manuscript:

ASA24	Automated Self-Assessment 24-H Dietary Recall
CC	Colorectal Cancer
EFNEP	Expanded Food and Nutrition Education Program
ESI	Eat Smart Idaho
FBI	Foodborne Illness
FFY	Federal Fiscal Year
FPAQ	Food and Physical Activity Questionnaire
GED	General Equivalency Diploma
HD	Heart Disease
HTN	Hypertension
NIFA	National Institute of Food and Agriculture
OB	Obesity
ONB	Optimal Nutrition Behaviors
OST	Osteoporosis
PSE	Policy, Systems, and Environmental
SNAP-Ed	Supplemental Nutrition Assistance Program Education
STK	Stroke
T2D	Type 2 Diabetes
USDA	United States Department of Agriculture
