# Supplementary material for: Defining Optimal Nutrition Behaviors to Determine Benefit–Cost Ratio of Federal Nutrition Education Programs
Source: Nutrients. 2025 Sep 27;17(19):3076. doi: 10.3390/nu17193076 (PMC12525610; doi:10.3390/nu17193076)
Supplement: Supplementary file 1 [file nutrients-17-03076-s001.zip › nutrients-3820631-supplementary material S1.pdf]

CBA - Adult Entry Form

Eat Smart Idaho
UNIVERSITY OF IDAHO EXTENSION

Date____________________________________

Name____________________________________

Address__________________________________

City_____________________________ **Zip**______

County ________________________________

Phone ________________________________

Age_________ **Check one**: ☐ Female ☐ Male

If female:

Pregnant? ☐ Yes ☐ No
Breastfeeding? ☐ Yes ☐ No

Check the ethnicity you identify with:

☐ Hispanic/Latino ☐ non-Hispanic/non-Latino

Check the race category you identify with:
(you may check more than one)

- ☐ American Indian/Alaskan Native
- ☐ Asian
- ☐ Black or African American
- ☐ Native Hawaiian or other Pacific Islander
- ☐ White

Highest Grade Completed____________________

- ☐ Graduated High School or GED
- ☐ Some college
- ☐ Graduated 2 year college
- ☐ Graduated college
- ☐ Post Graduate

Where do you live? (check one)

- ☐ Farm/Rural
- ☐ Towns under 10,000 and rural non-farm
- ☐ Towns & cities 10,000 to 50,000
- ☐ Suburbs of cities over 50,000
- ☐ Central cities over 50,000

Programs that you and your family participate in:
(check all that apply)

- ☐ Free or reduced school lunch or breakfast
- ☐ FDPIR (Food Distribution – Indian Reservations)
- ☐ Head Start (CPCD)
- ☐ Food Stamps (EBT)
- ☐ TAFI (Temporary Assistance for Families--TANF)
- ☐ TEFAP (Commodities)
- ☐ WIC
- ☐ Other ______________________________

Monthly Household Cash Income $____________

Household Members:
(list ages of children who live with you)

Age

_________ _________
_________ _________
_________ _________
_________ _________
_________ _________

Number of other adults in household ________

Where was this class taught?

- ☐ Adult education/training center
- ☐ Community Action Partnership
- ☐ Emergency food assistance site/food bank/pantry
- ☐ Food Stamp Office/ Health & Welfare
- ☐ Head Start/Early Head Start
- ☐ Health Clinic/Public or Community Health Center
- ☐ Public Housing
- ☐ School (K-12)
- ☐ Shelter or Transitional Housing
- ☐ WIC Office
- ☐ Working Solutions
- ☐ Other ______________________________

Employee/Office Use Only

Nutrition Advisor:____________________________________ ASA24 ID______________

Lesson Type: ☐ Group ☐ Individual ☐ Both Program: ☐ EFNEP ☐ SNAP-Ed

Group Instruction: Name of Group________________________________

State Office Use Only

ID Number: ________________

CBA - Adult Exit Form

Date ______________________________

Name ______________________________

Address ______________________________

City ____________________Zip ________

County ______________________________

Phone ______________________________

Email ______________________________

If female:

Pregnant? ☐ Yes ☐ No

Breastfeeding? ☐ Yes ☐ No

Programs that you and your family participate in (check all that apply):

☐ Free or reduced school lunch or breakfast
☐ FDPIR (Food Distribution – Indian Reservations)
☐ Head Start (CPCD)
☐ Food Stamps (EBT)
☐ TAFI (Temporary Assistance for Families – TANF)
☐ TEFAP (Commodities)
☐ WIC
☐ Other ______________________________

I learned: ______________________________

____

____

____

Please mark if you are interested in future classes, newsletters, etc.:

☐ Yes ☐ No

Office Use Only

Lesson	Date	Time Spent Teaching
Choosing to Move Throughout the Day		
Choosing More Fruits and Vegetables		
Fix it Safe		
Plan: Know What's for Dinner		
Shop: Get the Best for Less		
Shop for Value, Check the Facts		
Other:		

Office Use Only

Nutrition Advisor's Name: ______________________________

Group Name: ____________________ ASA24 ID____________

Total Number of Lessons: __________

Total Number of Sessions (Contacts): ____________

Total Number of Hours: ______________

Lesson Type: ☐ Group ☐ Individual ☐ Both

Termination Reason:

☐ Educational Objectives Met (Graduate)
☐ Returned to School
☐ Took Job
☐ Family Concerns
☐ Staff Vacancy
☐ Moved
☐ Lost interest
☐ Other Obligation
☐ Lost Contact with Client
☐ Other ______________________________
