# Supplementary material for: Defining Optimal Nutrition Behaviors to Determine Benefit–Cost Ratio of Federal Nutrition Education Programs
Source: Nutrients. 2025 Sep 27;17(19):3076. doi: 10.3390/nu17193076 (PMC12525610; doi:10.3390/nu17193076)
Supplement: Supplementary file 1 [file nutrients-17-03076-s001.zip › nutrients-3820631-supplementary material S2.pdf]

Eat Smart Idaho
UNIVERSITY OF IDAHO EXTENSION

☐ ENTRY ☐ EXIT

NAME

DATE / /

Food & Physical Activity Questionnaire

Please mark the response that **best** describes how you **usually** do things.

1. How many **times a day** do you eat fruit?

Examples of fruits are apples, bananas, oranges, grapes, raisins, melon and berries. Include fresh, frozen, dried, or canned fruit. ***Do not include juice.***

- ☐ I rarely eat fruit
- ☐ Less than 1 time a day (a couple times a week)
- ☐ 1 time a day
- ☐ 2 times a day
- ☐ 3 times a day
- ☐ 4 or more times a day

2. How many **times a day** do you eat vegetables?

Examples of vegetables are green salad, corn, green beans, carrots, potatoes, greens, and squash. Include fresh, canned and frozen vegetables. ***Do not count french fries, potato chips or rice.***

- ☐ I rarely eat vegetables
- ☐ Less than 1 time a day (a couple times a week)
- ☐ 1 time a day
- ☐ 2 times a day
- ☐ 3 times a day
- ☐ 4 or more times a day

3. Over the last week, **how many days** did you eat red and orange vegetables?

Examples of red or orange vegetables are tomatoes, red peppers, carrots, sweet potatoes, winter squash, and pumpkin.

- ☐ I did not eat red and orange vegetables
- ☐ 1 day a week
- ☐ 2 days a week
- ☐ 3 days a week
- ☐ 4 days a week
- ☐ 5 days a week
- ☐ 6 or 7 days a week

4. Over the last week, **how many days** did you eat dark green vegetables?

Examples of dark green vegetables are broccoli, spinach, dark green lettuce, turnip greens, or mustard greens.

- ☐ I did not eat dark green vegetables
- ☐ 1 day a week
- ☐ 2 days a week
- ☐ 3 days a week
- ☐ 4 days a week
- ☐ 5 days a week
- ☐ 6 or 7 days a week

5. How often do you drink regular sodas (not diet)?

- ☐ Never
- ☐ 1 – 3 times a week
- ☐ 4 – 6 times a week
- ☐ 1 time a day
- ☐ 2 times a day
- ☐ 3 times a day
- ☐ 4 or more times a day

6. How often do you drink fruit punch, fruit drinks, sweet tea or sports drinks?

- ☐ Never
- ☐ 1 – 3 times a week
- ☐ 4 – 6 times a week
- ☐ 1 time a day
- ☐ 2 times a day
- ☐ 3 times a day
- ☐ 4 or more times a day

There is more on the next page ➧

7. In the past week, <u>how many days</u> did you exercise for at least 30 minutes?

This includes things like jogging, playing soccer, and doing fitness or dance classes, or exercise videos. <u>This 30 minutes could be all at once or 10 minutes or more at a time.</u> *Do not count housework, taking care of your kids, or walking from place to place.*

- ☐ 0 days
- ☐ 1 day
- ☐ 2 days
- ☐ 3 days
- ☐ 4 days
- ☐ 5 days
- ☐ 6 days
- ☐ 7 days

8. In the past week, <u>how many days</u> did you do workouts to build and strengthen your muscles?

This includes things like lifting weights and doing push-ups, sit-ups or planks.

- ☐ 0 days
- ☐ 1 day
- ☐ 2 days
- ☐ 3 days
- ☐ 4 days
- ☐ 5 days
- ☐ 6 days
- ☐ 7 days

9. How often do you make small changes on purpose to be more active?

This includes things like walking instead of driving, getting off the bus one stop early, doing a few minutes of exercise, or moving around instead of sitting while watching TV.

- ☐ Never
- ☐ Rarely (about 20% of the time)
- ☐ Sometimes (about 40% of the time)
- ☐ Often (about 60% of the time)
- ☐ Usually (about 80% of the time)
- ☐ Always

10. How often do you wash your hands with soap and running water before preparing food?

- ☐ Never
- ☐ Rarely (about 20% of the time)
- ☐ Sometimes (about 40% of the time)
- ☐ Often (about 60% of the time)
- ☐ Usually (about 80% of the time)
- ☐ Always

11. After cutting raw meat or seafood, how often do you wash all items and surfaces that came in contact with these foods?

- ☐ Never
- ☐ Rarely (about 20% of the time)
- ☐ Sometimes (about 40% of the time)
- ☐ Often (about 60% of the time)
- ☐ Usually (about 80% of the time)
- ☐ Always

12. How often do you thaw frozen food on the counter or in the sink at room temperature?

- ☐ Never
- ☐ Rarely (about 20% of the time)
- ☐ Sometimes (about 40% of the time)
- ☐ Often (about 60% of the time)
- ☐ Usually (about 80% of the time)
- ☐ Always

13. How often do you use a meat thermometer to see if meat is cooked to a safe temperature?

- ☐ Never
- ☐ Rarely (about 20% of the time)
- ☐ Sometimes (about 40% of the time)
- ☐ Often (about 60% of the time)
- ☐ Usually (about 80% of the time)
- ☐ Always

14. In the past month, how often did you eat less than you wanted so there was more food for your family?

- ☐ Never
- ☐ Rarely (about 20% of the time)
- ☐ Sometimes (about 40% of the time)
- ☐ Often (about 60% of the time)
- ☐ Usually (about 80% of the time)
- ☐ Always

There is more on the next page ➧

15. **In the past month, how often did you not have money or another way to get enough food for your family (such as SNAP, WIC, or a food pantry)?**

☐ Never
☐ Rarely (about 20% of the time)
☐ Sometimes (about 40% of the time)
☐ Often (about 60% of the time)
☐ Usually (about 80% of the time)
☐ Always

16. **How many <u>days a week</u> do you cook dinner (your main meal) at home?**

☐ I rarely cook dinner at home
☐ 1 day a week
☐ 2 days a week
☐ 3 days a week
☐ 4 days a week
☐ 5 days a week
☐ 6 or 7 days a week

17. **How often do you compare food prices to save money?**

☐ Never
☐ Rarely (about 20% of the time)
☐ Sometimes (about 40% of the time)
☐ Often (about 60% of the time)
☐ Usually (about 80% of the time)
☐ Always

18. **How often do you plan your meals before you shop for groceries?**

☐ Never
☐ Rarely (about 20% of the time)
☐ Sometimes (about 40% of the time)
☐ Often (about 60% of the time)
☐ Usually (about 80% of the time)
☐ Always

19. **How often do you look in the refrigerator or cupboard to see what you need before you go shopping?**

☐ Never
☐ Rarely (about 20% of the time)
☐ Sometimes (about 40% of the time)
☐ Often (about 60% of the time)
☐ Usually (about 80% of the time)
☐ Always

20. **How often do you make a list before going shopping?**

☐ Never
☐ Rarely (about 20% of the time)
☐ Sometimes (about 40% of the time)
☐ Often (about 60% of the time)
☐ Usually (about 80% of the time)
☐ Always

21. **How often do you use the Nutrition Facts Label when deciding to buy a food product?**

Think about the Nutrition Facts label on many food products that list the amounts of things like calories, fat, cholesterol, vitamins and minerals in the product.

☐ Never
☐ Rarely (about 20% of the time)
☐ Sometimes (about 40% of the time)
☐ Often (about 60% of the time)
☐ Usually (about 80% of the time)
☐ Always

Employee/Office Use Only

ASA 24 ID_______________

Group Name _________________________

This work and material is supported and funded by USDA's Supplemental Nutrition Assistance Program-SNAP and the Expanded Food and Nutrition Education Program from the USDA National Institute of Food and Agriculture. USDA and University of Idaho are equal opportunity providers.
