# Supplementary material for: Defining Optimal Nutrition Behaviors to Determine Benefit–Cost Ratio of Federal Nutrition Education Programs
Source: Nutrients. 2025 Sep 27;17(19):3076. doi: 10.3390/nu17193076 (PMC12525610; doi:10.3390/nu17193076)
Supplement: Supplementary file 1 [file nutrients-17-03076-s001.zip › nutrients-3820631-supplementary material S3.pdf]

Eat Smart Idaho PSE Programs Survey

Name______________________________ Date_______________________

This survey asks about your familiarity with various Eat Smart Idaho programs. Please mark the appropriate box to indicate your answer to each question below.

1. **Please check any of the food pantries below where you have gotten food in the last year.**

NORTHERN IDAHO

☐ Bonner Community Food Center	☐ J-K Good Samaritan Food Bank	☐ Potlatch Food Bank
☐ Boys & Girls Club Food Pantry – Coeur d'Alene	☐ Lake City Community Food Pantry	☐ Real Life Ministries Pantry
☐ Canvas Church Food Pantry	☐ LCSC Warrior Pantry	☐ Seventh Day Adventist Food Bank
☐ CAP Food Bank – Coeur d'Alene	☐ Lifeline Food Bank	☐ The Altar Food Bank
☐ CAP Food Bank - Lewiston	☐ Moscow Food Bank	☐ Troy Food Bank
☐ Christ the King Lutheran Church Food Bank	☐ Nez Perce Tribe Emergency Food Bank	☐ Vandal Food Pantry Cabinet
☐ Coeur d'Alene Tribe Food Distribution Services	☐ Post Falls Food Bank	☐ Venture Alternative High School

SOUTHERN IDAHO

☐ Salvation Army		
☐ St. Vincent de Paul		

SOUTH-CENTRAL IDAHO

☐ Canyon Ridge High School	☐ Mary and Martha Pantry	☐ Robert Stuart Middle School
☐ Cassia Alternative High School	☐ Open Hearts Food Pantry	☐ The Mustard Seed
☐ Hope for the Hungry		

EASTERN IDAHO

☐ Benny's Pantry-ISU	☐ Salvation Army	☐ St Vincent de Paul
☐ Blackfoot Community Pantry	☐ Preston Community Pantry	☐ Upper Valley Pantry
☐ Community Food Basket		

Eat Smart Idaho PSE Programs Survey

2. If you checked any of the food pantries in question 1, did you notice these MyPlate Signs at the pantry?

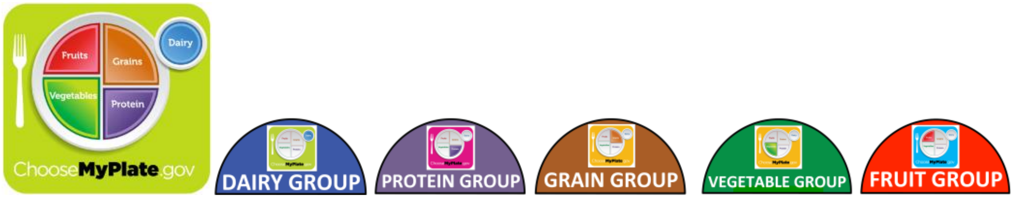

☐ Yes

☐ No

☐ I cannot remember

3. Have you noticed a healthy food drive flyer in your community, similar to the one below?

☐ Yes

☐ No

☐ I cannot remember

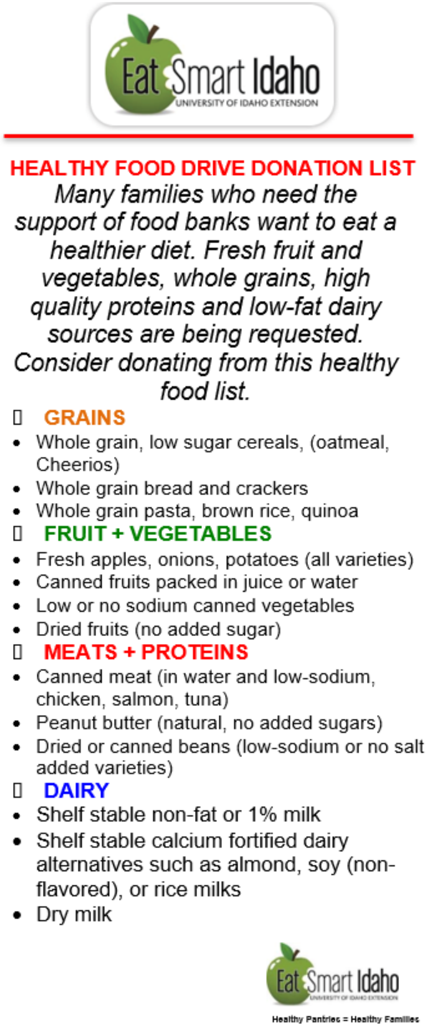
Eat Smart Idaho
UNIVERSITY OF IDAHO EXTENSION

HEALTHY FOOD DRIVE DONATION LIST

Many families who need the support of food banks want to eat a healthier diet. Fresh fruit and vegetables, whole grains, high quality proteins and low-fat dairy sources are being requested. Consider donating from this healthy food list.

GRAINS
- Whole grain, low sugar cereals, (oatmeal, Cheerios)
- Whole grain bread and crackers
- Whole grain pasta, brown rice, quinoa

FRUIT + VEGETABLES
- Fresh apples, onions, potatoes (all varieties)
- Canned fruits packed in juice or water
- Low or no sodium canned vegetables
- Dried fruits (no added sugar)

MEATS + PROTEINS
- Canned meat (in water and low-sodium, chicken, salmon, tuna)
- Peanut butter (natural, no added sugars)
- Dried or canned beans (low-sodium or no salt added varieties)

DAIRY
- Shelf stable non-fat or 1% milk
- Shelf stable calcium fortified dairy alternatives such as almond, soy (non-flavored), or rice milks
- Dry milk

Eat Smart Idaho
Healthy Pantries = Healthy Families

Eat Smart Idaho PSE Programs Survey

4. Do you care for children that attend any of the following schools? Please check all that apply.

NORTHERN IDAHO

☐ Atlas Elementary	☐ John Brown Elementary	☐ Seltice Elementary
☐ Borah Elementary	☐ Mullan Trail Elementary	☐ Skyway Elementary
☐ Bryan Elementary	☐ Northwest Expedition Academy	☐ Sorenson Magnet School
☐ Dalton Elementary	☐ Ponderosa Elementary	☐ West Ridge Elementary
☐ Fernan Elementary	☐ Prairie View Elementary	☐ Winton Elementary
☐ Hayden Meadows Elementary	☐ Ramsey Elementary	

SOUTHERN IDAHO

☐ Marsing Elementary	☐ Marsing Middle School	☐ Marsing High School
☐ Wilder Elementary	☐ Wilder Middle School	☐ Wilder High School
☐ Emmett Middle School		

SOUTH-CENTRAL IDAHO

☐ Hansen School	☐ Murtaugh School	☐ South Hills Middle School
☐ Hollister Elementary School	☐ Popplewell Elementary	☐ Summit Elementary School
☐ Mountain View Elementary	☐ Robert Stuart Middle School	☐ Valley School

EASTERN IDAHO

☐ Fort Hall Elementary School	☐ Independence HS	☐ Snake River MS
☐ Grace Elementary School	☐ Falls Valley Elementary	☐ Thatcher Elementary School
☐ Harwood Elementary School	☐ Robert's Elementary	☐ Harold B. Lee Elementary

Eat Smart Idaho PSE Programs Survey

5. Have you noticed any of the following posters in your community?

☐ Yes
☐ No
☐ I cannot remember

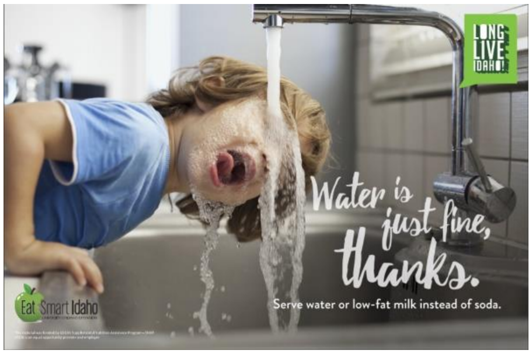

☐ Yes
☐ No
☐ I cannot remember

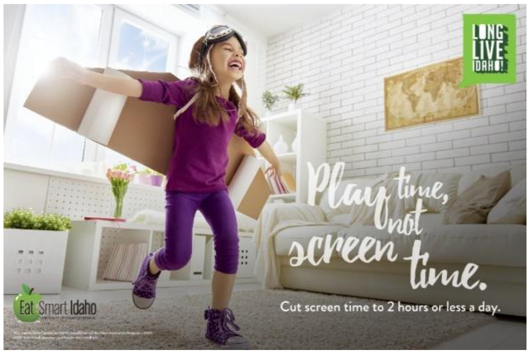

☐ Yes
☐ No
☐ I cannot remember

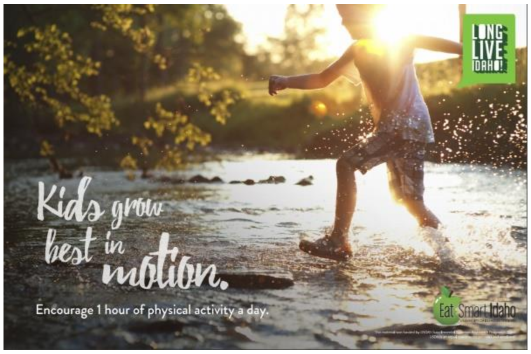

☐ Yes
☐ No
☐ I cannot remember

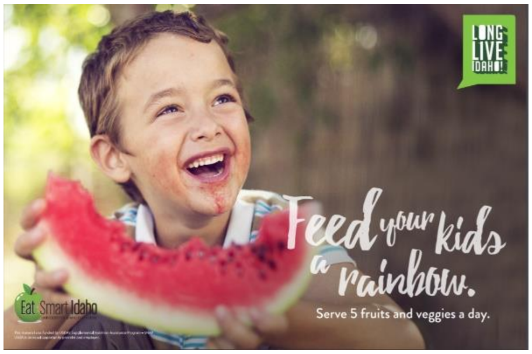

Employee/Office Use Only

ASA-24 ID____________________________ Group Name____________________________________
